# Modelling short‐rotation coppice and tree planting for urban carbon management – a citywide analysis

**DOI:** 10.1111/1365-2664.12491

**Published:** 2015-07-16

**Authors:** Nicola McHugh, Jill L. Edmondson, Kevin J. Gaston, Jonathan R. Leake, Odhran S. O'Sullivan

**Affiliations:** ^1^ Department of Animal and Plant Sciences University of Sheffield Alfred Denny Building Western Bank Sheffield S10 2TN UK; ^2^ Environment and Sustainability Institute University of Exeter Penryn Cornwall TR10 9FE UK

**Keywords:** ecosystem services, GIS model, land‐use, short‐rotation coppice, urban biomass carbon, urban ecosystems, wood biofuel

## Abstract

1. The capacity of urban areas to deliver provisioning ecosystem services is commonly overlooked and underutilized. Urban populations have globally increased fivefold since 1950, and they disproportionately consume ecosystem services and contribute to carbon emissions, highlighting the need to increase urban sustainability and reduce environmental impacts of urban dwellers. Here, we investigated the potential for increasing carbon sequestration, and biomass fuel production, by planting trees and short‐rotation coppice (SRC), respectively, in a mid‐sized UK city as a contribution to meeting national commitments to reduce CO_2_ emissions.
2. Iterative GIS models were developed using high‐resolution spatial data. The models were applied to patches of public and privately owned urban greenspace suitable for planting trees and SRC, across the 73 km^2^ area of the city of Leicester. We modelled tree planting with a species mix based on the existing tree populations, and SRC with willow and poplar to calculate biomass production in new trees, and carbon sequestration into harvested biomass over 25 years.
3. An area of 11 km^2^ comprising 15% of the city met criteria for tree planting and had the potential over 25 years to sequester 4200 tonnes of carbon above‐ground. Of this area, 5·8 km^2^ also met criteria for SRC planting and over the same period this could yield 71 800 tonnes of carbon in harvested biomass.
4. The harvested biomass could supply energy to over 1566 domestic homes or 30 municipal buildings, resulting in avoided carbon emissions of 29 236 tonnes of carbon over 25 years when compared to heating by natural gas. Together with the net carbon sequestration into trees, a total reduction of 33 419 tonnes of carbon in the atmosphere could be achieved in 25 years by combined SRC and tree planting across the city.
5. *Synthesis and applications*. We demonstrate that urban greenspaces in a typical UK city are underutilized for provisioning ecosystem services by trees and especially SRC, which has high biomass production potential. For urban greenspace management, we recommend that planting SRC in urban areas can contribute to reducing food–fuel conflicts on agricultural land and produce renewable energy sources close to centres of population and demand.

## Abstract 2

We demonstrate that urban greenspaces in a typical UK city are underutilized for provisioning ecosystem services by trees and especially SRC, which has high biomass production potential. For urban greenspace management, we recommend that planting SRC in urban areas can contribute to reducing food–fuel conflicts on agricultural land and produce renewable energy sources close to centres of population and demand.

## Introduction

Urban populations depend on rural areas to supply essential provisioning ecosystem services including food, fibres, wood and water, and it is often assumed that urban areas are unable to make any significant contribution to such services. However, urban greenspaces deliver a variety of supporting, regulating and cultural ecosystem services (Davies *et al*. 2011a; Gómez‐Baggethun *et al*. 2013; Nowak *et al*. 2013a), including high species richness (McKinney 2008), improved psychological well‐being (Fuller *et al*. 2007), reduced stormwater run‐off and air pollution interception (Sæbø *et al*. 2012). Better management of urban greenspace to deliver multiple ecosystem services has the potential to simultaneously enhance the quality of life for city dwellers and the sustainability of urban areas (Davies *et al*. 2011a). Despite such evidence, the potential for urban greenspaces to deliver provisioning ecosystem services such as biomass fuel and timber, and regulating services, such as carbon storage, has received little attention in the UK. Consequently, the extent to which tree planting can contribute to CO_2_ emissions reduction targets through carbon sequestration into biomass or through biofuel substitution for fossil fuels in UK cities remains unclear.

Urban areas are expanding globally, with urban populations increasing fivefold from 0·8 to 3·6 billion between 1950 and 2011 (United Nations 2012), and these areas disproportionately contribute to global anthropogenic CO_2_ emissions (UN‐Habitat 2011). The UK is committed to reducing national CO_2_ emissions by 80% of 1990 values by 2050 (UK Parliament 2008), requiring a major reduction in fossil fuel use. Maximizing local energy production and increasing carbon sequestration into biomass will undoubtedly be among the range of solutions required to achieve this ambitious goal.

Appropriately planned and managed, urban greenspaces could deliver increases in specific ecosystem services such as carbon storage in trees, as seen in urban tree planting in the UK (Díaz‐Porras, Gaston & Evans 2014) and USA (Nowak *et al*. 2013b; McPherson & Kendall 2014). In Leicester, a typical UK city, trees account for 97·3% of carbon stored in above‐ground vegetation (Davies *et al*. 2011b) confirming their importance in ecosystem carbon storage. Urban tree planting has been promoted to enhance multiple ecosystems service benefits (Roy, Byrne & Pickering 2012) including: air pollution interception (Sæbø *et al*. 2012); noise reduction (Roy, Byrne & Pickering 2012); enhanced stormwater infiltration (Stovin, Jorgensen & Clayden 2008); reduced building energy use for summer cooling (Rahman, Armson & Ennos 2014) and recreation, aesthetic and cultural benefits (Kaplan 2007).

Larger greenspace areas may have the potential for growing short‐rotation coppice (SRC), a system for woody biomass production. SRC refers to any woody species (typically high‐yielding species such as poplar and willow), which is managed in a coppice system, typically harvested every 3–5 years and normally grown as a biofuel crop (Aylott *et al*. 2008, 2010). This can contribute to the UK Government target for 15% of energy to come from renewable sources by 2020 (DECC 2011).

Despite the large areas of greenspace within towns and cities, current UK SRC guidance is exclusively focussed on agricultural land (Natural England, 2013a). However, constraints identified in this guidance do not necessarily preclude SRC in urban areas, indeed the urban fringe was identified as particularly suited to such crops in an earlier report (British BioGen 1996). Many of the recommendations for increasing biodiversity within SRC patches (Rowe, Street & Taylor 2009) are achievable in urban areas, including plantations with large edge to interior ratio, small plot sizes and blocks of SRC interspersed with other habitats.

The fragmented heterogeneous structure of urban landscapes due to division of land into small patches under different ownership, management and diverse usage (Luck & Wu 2002) is exemplified by domestic gardens which account for 22–27% of greenspace in UK urban areas (Loram *et al*. 2007). High‐resolution spatial data are overcoming the problem of assessing the ecosystem services provided by such small land parcels (Davies *et al*. 2013).

Here, we assess the potential to increase carbon sequestration in trees and harvested SRC biomass in a typical UK city. On the basis of previous estimates, the contribution of SRC biomass to heat municipal buildings and homes and the reduction in CO_2_ emissions achieved by this biomass substituting for natural gas heating homes is assessed. Wood‐fuel biomass boilers have gained increasing importance in municipal heating systems and schools (The Carbon Trust, 2012); however, there has been surprisingly little research to date on biomass fuel production in urban areas (but see Nielsen & Møller 2008; MacFarlane 2009; Strohbach *et al*. 2012; McPherson & Kendall 2014; Zhao *et al*. 2014).

We developed modelling tools to address the specific challenges of simulating tree and SRC growth to ensure that the modelled trees could be fitted into the existing landscape and continue to do so as they grew. The tree‐planting model identified suitable sites for planting and was designed to maintain the existing diversity of tree species within the urban study area, based on recent surveys of trees in Leicester (Davies *et al*. 2011b), matching tree size at maturity to the greenspace patch sizes.

## Materials and methods

### Study Area

This study focused on Leicester (52°38′N, 1°08′W), a typical mid‐sized city in central England with a population of around 310 000, and annual CO_2_ emissions of 478 000 tonnes of carbon (Leicester City Council, 2012). The 73‐km^2^ city area has a densely developed urban core, beyond which are suburbs, with built development reaching the city boundary in the east and west and small peri‐urban areas to the north and south. The annual daily mean temperature range is 1·7–21·3 °C with 606‐mm annual rainfall (Met Office 2012).

Land ownership was divided into private (land within the boundary of private dwellings, identified through MasterMap) (Ordnance Survey 2008), public (land owned by Leicester City Council) or mixed‐land ownership (areas belonging to business or private individuals and land where ownership was undetermined). Land cover was derived from the LandBase data set (Infoterra 2006), which identifies eight land cover classes: bare ground, inland water, artificial surface, buildings, herbaceous (mainly grassland), shrub, tall shrub and trees (0·25 m^2^ resolution). Only areas categorized as herbaceous or bare ground were considered suitable for tree or SRC planting in our models, with shrub, tall shrub and tree land cover, and areas currently under artificial surface or buildings, excluded.

### Mixed‐Species Tree‐Planting Models

Separate mixed‐species tree‐planting models were developed to apply to private land (Fig. S1, Supporting information) and public and mixed ownership land (Fig. S2), as the small land parcel size in private land necessitated the use of a separate model. The two GIS models (ESRI ArcInfo 10, ModelBuilder) iteratively planted trees allowing planting restriction to be applied to avoid areas deemed unsuitable (Table S2).

Building on an approach developed by Wu, Xiao & McPherson (2008) for Los Angeles, the models analysed the current landscape in order to predict the ability to accommodate trees, including allowing for tree growth over 25 years, a modelling time span that reflects the use of current climate information and is consistent with recent studies of effects of peri‐urban trees on air quality (Kroeger *et al*. 2014). Combining data from the tree survey carried out by Davies *et al*. (2011b) and a garden tree survey using the same methodology (data available from the Dryad Digital Repository: http://dx.doi.org/10.5061/dryad.j25t0; McHugh *et al*. 2015), over 1300 trees in Leicester were identified and diameter at breast height (d.b.h.) measured. Those species with more than one individual (68 species) were included in the tree‐planting models.

Mature crown diameter values of large (15 m) and small (5 m) species within the tree population were incorporated into the models reducing the risk of overplanting the landscape, replicating the species and size heterogeneity of the current urban forest and developing more realistic carbon storage values than could be achieved with a single species planting model. Trees planted were modelled on whips [<2 cm diameter, 100–200 cm height (ENA 2010; Forestry Commission 2010)], with a mean diameter planting size of 0·53 cm determined from Willoughby *et al*. (2007).

Minimum distance restrictions from impervious surfaces (measured from trunk) of 6 or 2 m for large and small trees, respectively, were applied. These values were determined by combining root spread values of tree species from the local population, expressed as a percentage of mean crown diameter (Gruffydd 1987; Hodge & White 1990; RHS 2014), together with existing distance guidelines to minimize damage to nearby buildings, roads and paths (Gasson & Cutler 1998) (Table S1). Such guidelines have economic relevance – in the London Borough of Hackney, UK, 40% of trees removed from 2002 to 2007 were a result of insurance claims for tree‐related property damage (LAEC, 2007).

The private ownership model (Fig. S1) in domestic gardens had a minimum area requirement of 9 m^2^ for large trees and 2 m^2^ for small trees with no overlap of existing or newly planted tree canopies stipulated. The model continued searching for planting sites until the number of trees planted in each cycle was <10 large or 1000 small trees, determined to balance search time with additional trees planted. The separate modelling approach applied to public and mixed ownership land was designed to maximize planting in larger spaces (Fig. S2). This model incorporated a single cycle of large tree planting followed by the removal of unsuitably sited trees, that is where mature canopies would extend beyond the suitable planting area. The final stage identified sites that could still accommodate small trees and filled gaps within the planting scheme. Identical tree size and minimum distances to buildings, roads and paths were used in private, and public and mixed ownership models.

Urban‐specific mortality rates for newly planted trees (0–3 years) of 10%, and for established trees (4–25 years) of 6%, were applied (Gilbertson & Bradshaw 1990; Nowak, McBride & Beatty 1990; Bradshaw, Hunt & Walmsley 1995; Nowak, Kuroda & Crane 2004; LAEC 2007). A replanting phase (5% trees aged 0–3 years, 3% trees aged 4–25 years) then occurred outside the spatial modelling environment. The number and size of trees removed from the models through annual mortality events was calculated in order to quantify carbon removed from the study area.

Annual tree growth rates were taken from the literature and applied for 25 years to planted trees. Species‐specific rates were used when available, or else genus or family specific rates were used (see Table S3), with growth rates of urban trees in the same geographic region as the study site used preferentially. Linear growth rates were applied as growth is unlikely to slow in the first 25 years (Strohbach *et al*. 2012). The above‐ground biomass of trees was calculated annually using species‐ and genus‐specific allometric equations (see Table S4), and a biomass‐to‐carbon conversion factor of 0·46 for broadleaf and 0·42 for coniferous species was used to determine carbon content (Milne & Brown 1997). The use of generalized equations (up to eight annual growth rates and six allometric biomass equations) minimized variability, an issue identified by McHale *et al*. (2009) when applying non‐urban equations to urban trees. To compare the mixed‐species models, the maximum possible increase in carbon storage by tree planting was estimated using the fastest growing large (*Eucalyptus gunnii* Hook. F.) and small trees (*Populus tremula* L.) in our data base (Table S3).

### SRC Model

Potential SRC yield for combined willow and poplar plantings was calculated based on regional mean values based on Agricultural Land Classification (ALC) (Aylott *et al*. 2010). As no yield value was provided for the ALC ‘urban’ category, the yield for lowest quality (category 5) land, of 10·3 oven‐dry tonnes (odt) ha^−1^ year^−1^, was used. This is a conservative approach as citywide analysis of soil properties in Leicester found that in most greenspaces, the soil quality matches or exceeds that of agricultural land (Edmondson *et al*. 2011, 2012, 2014). A series of spatial restriction criteria, based on UK Energy Crop Scheme guidance (Natural England 2013b) and findings of biofuels research (Renewable Fuels Agency 2008; Aylott *et al*. 2010), was developed (Table S2) to identify suitable planting sites and the annual yield possible across the study area was calculated. The heating and fossil fuel offset potential of SRC yields were estimated (see Appendices S1 and S2) using published values for the biomass of wood chips required to heat a typical domestic house, municipal building or support a district heating scheme (Biomass Energy Centre 2014). The fossil fuel carbon savings of biomass substitution for natural gas was calculated using data on household gas consumption from DECC (2013), and the net fossil fuel savings relative to natural gas provided by SRC wood chips, taking into account fossil fuel costs of harvesting, transport, chipping, drying and distribution (Defra 2009).

### Comparison of Tree and SRC Planting Model Outputs

The increase in carbon sequestration resulting from the two carbon management approaches, the mixed‐species tree planting and SRC models was compared at years 10 and 25 to the above‐ground carbon stocks of the existing tree population of the study area. In addition, a combined management approach giving priority to SRC on all suitable land followed by the application of the mixed‐species tree‐planting model to remaining suitable sites was employed to maximize effects of carbon management.

## Results

The tree‐planting models identified an area of 11 km^2^ suitable for planting, 86·5% of which was in public or mixed ownership, and only 13·5% was in private gardens (Table 1). Nonetheless, gardens were found to be able to accommodate 70 000 additional, mainly small, trees. Over 25 years, these trees could enhance carbon stocks by six times the current amounts in above‐ground herbaceous vegetation in the areas of gardens allocated to tree planting (Tables 1 and 2). This is a higher proportional increase in carbon storage than that found by the model of public or mixed ownership land, which projects a doubling of carbon storage over 25 years in areas of herbaceous vegetation allocated to the planting of a total of 220 000 trees. Most of these trees were of species too large for gardens once fully grown and therefore were planted at a lower density than the small trees.

**Table 1:** Area of greenspace suitable for tree planting or short‐rotation coppice (SRC), and estimates of the above‐ground carbon stocks in vegetation in these areas

Greenspace management approach	Land ownership	Total greenspace area under herbaceous vegetation (m^2^)	Area of herbaceous greenspace suitable for management approach^a^		Current above‐ground carbon in area suitable for management approach^b^ (tonnes)
			m^2^	%	
Tree planting	Public	12 647 614	3 096 813	47·5	464·522
	Mixed	6 524 299	6 475 435	51·2	906·561
	Private	8 402 581	1 494 506	17·8	209·231
	All	27 574 494	11 066 754	40·1	1580·314
SRC establishment	Public	12 647 614	1 710 878	26·2	256·632
	Mixed	6 524 299	4 154 263	32·8	581·597
	All	19 171 913	5 865 141	30·6	838·229
Combined	All	27 574 494	11 066 754	40·1	1580·314

a Suitable areas were identified after spatial restriction criteria were applied (areas covered in shrubs or trees were excluded).

b See Davies *et al*. (2011b) for further details.

**Table 2:** Potential increase in carbon sequestration into live trees and harvested short‐rotation coppice (SRC) biomass over 25 years, and potential carbon offsetting by SRC biomass substitution for natural gas in domestic heating and tree planting

Greenspace management approach	Carbon (tonnes) sequestered into newly planted trees or harvested SRC biomass [carbon offset by SRC, and under combined management the total carbon sequestered plus offset for tree planting plus SRC]		
	Year 0^a^	Year 10	Year 25
Tree planting			
Public ownership	0·286	167·377	1024·389
Mixed ownership	0·512	294·266	1821·020
Private ownership	7·226	249·024	1337·278
Total	8·024	710·667	4182·687
SRC establishment			
Public ownership	0	8383·302 [3411·341]	20958·256 [8528·354]
Mixed ownership	0	20355·889 [8283·238]	50889·722 [20708·096]
Total	0	28739·191 [11694·580]	71847·978 [29236·450]
Combined management approach	7·726	29309·877 [12405·247]	74983·920 [33419·137]

a Year 0 values refer to imported carbon for tree‐planting establishment. The carbon import of SRC is assumed to be zero as establishment is from small cuttings.

Carbon storage increases resulting from applying the tree‐planting models are strongly influenced by the differing tree species compositions between land ownership classes. On domestic land, 23% of trees were fast‐growing *Cupressaceae* which over the 25‐year period individually sequestered *c. *96‐kg carbon (d.b.h. 33 cm). The species composition of trees found in public and mixed ownership land was more diverse and although the most common tree species have the potential to reach a large size, they often grow more slowly, for example *Fraxinus excelsior* L. with a d.b.h. of 14 cm at 25 years. Because of the initially small size and associated slow growth rates of many of the trees, the model projected a total increase in above‐ground carbon storage in biomass compared to herbaceous vegetation by only 2600–4200 tonnes over 25 years (Tables 1 and 2). However, as a consequence, we expect tree planting to supplement rather than to replace the existing herbaceous biomass. Carbon removed from the study area as a result of tree mortality over 25 years totalled 224 tonnes of carbon (private land ownership model) and 460 tonnes of carbon (public and mixed ownership model), giving a total removal of tree biomass of 684 tonnes. Although likely to be unacceptable from a biodiversity and aesthetic perspective (Roy, Byrne & Pickering 2012), maximizing carbon sequestration using the fastest growing large and small tree species (*E. gunnii* and *P. tremula*) indicated potential increased storage of 53 000 tonnes of carbon after 25 years – over 12 times greater than the projection from the model with multiple species (Table 2).

In comparison with tree planting, the SRC planting model projected much larger total biomass production of 71 848 tonnes across the city over 25 years, 20 958 tonnes of carbon being produced by SRC on public land and 50 889 tonnes of carbon on mixed ownership land (Table 2). These quantities are striking considering that the SRC model identified only 5·87 km^2^ (8% of the city) as suitable for planting, reflecting the high planting density and repeated harvesting of fast‐growing coppice biomass every 4 years which allows for rapid regrowth and associated conversion of atmospheric carbon to biomass.

Under the combined tree planting and SRC management, 73 400 tonnes of extra carbon could be captured by tree biomass and harvested SRC biomass (Tables 1 and 2) using 15% of the land area across Leicester. Total carbon removed by tree mortality in this case was estimated to be only 245 tonnes of carbon over 25 years.

**Figure 1:**
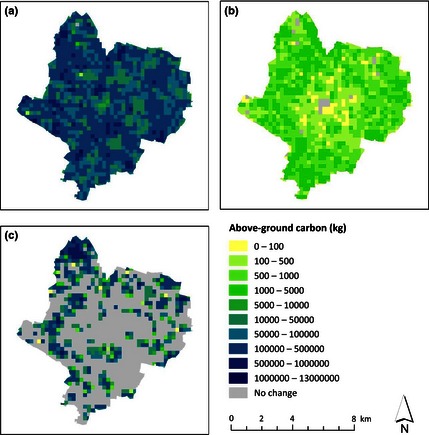
(a) Current total above‐ground carbon in 250 × 250 m grids across the city, (b) additional biomass carbon after 25 years predicted by the mixed‐species tree‐planting models and (c) carbon converted to harvested biomass over 25 years predicted by the short‐rotation coppice (SRC) model.

The spatial distribution of current above‐ground carbon in Leicester, together with projected 25‐year carbon conversion to live biomass (trees) and harvested biomass (SRC), is presented in Fig. 1. Current stocks of above‐ground carbon (Fig. 1a) average 3·16 kg m^−2^, with greatest storage corresponding with managed parkland and other large greenspaces, largely on the city outskirts. Under the tree‐planting approach (Fig. 1b), increases are rarely above 0·06 kg of carbon m^−2^ in the city centre after 25 years owing to lack of space for large trees. Outside the city centre, a higher proportion of land is suitable for tree planting, but our models show across the city above‐ground carbon stocks only increase by 0·04–3·20 kg m^−2^ after 25 years. Nonetheless, these increases should be viewed in the context of the already high biomass of vegetation in the city compared to the UK average above‐ground vegetation carbon density of 0·497 kg carbon m^−2^ (Milne & Brown 1997).

**Figure 2:**
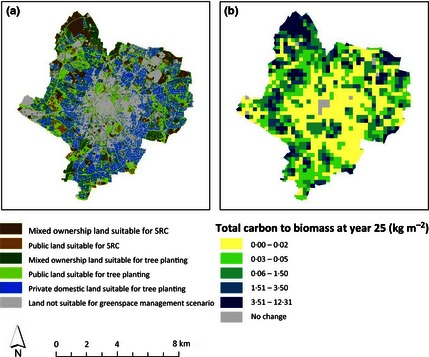
(a) Available urban greenspace suitable for management under the combined management approach and (b) total carbon assimilated both into above‐ground tree biomass, and harvested in short‐rotation coppice (SRC) over 25 years under the combined management approach in 250 × 250 m grids.

The areas suitable for SRC establishment are more limited and mainly in the urban fringes (Figs 1c and 2a). However, it is clear that where land is suitable for SRC, the quantity of carbon that can be fixed is far greater than that achievable by planting trees using a mixture of species similar to the existing urban tree population (Fig. 1b,c; Table 2).

The spatial distribution of potential carbon capture into trees and harvested SRC biomass production (Fig. 2b) clearly identifies areas, primarily on the city margins, with the greatest opportunities for a change in management. These are larger patches of public parks, undeveloped greenspace and brownfield sites near to industrial zones. The largest increases are due primarily to SRC, but enhancement of carbon stocks can take place across most of the city through utilizing small patches of urban greenspace for tree planting.

Based on our modelled SRC biofuel production potential across the city, averaging these yields over 25 years, could supply energy to 30 municipal buildings, or 52 district heating schemes (common in northern Europe and well suited to densely populated urban areas) (Biomass Energy Centre 2014). Using data from an award‐winning scheme in Barnsley, UK (Barnsley Metropolitan Borough Council 2006), the SRC biomass could support district heating of over 4200 flats, comprising 3% of households in Leicester. Domestic use of woodchip biofuel from SRC for heating would allow 1566 households to each avoid emissions of 746·7 kg carbon year^−1^ compared to the use of fossil fuel natural gas (Defra 2009), potentially avoiding 29 236 tonnes of fossil fuel carbon release over 25 years (Table 2). Together with the carbon sequestration into trees, additional to pre‐existing herbaceous vegetation, a total reduction of 33 419 tonnes of carbon in the atmosphere could be achieved in 25 years by combined SRC and tree planting across the city (Table 2).

## Discussion

The analysis presented here highlights the potential for enhanced carbon storage and mitigation of anthropogenic CO_2_ emissions by tree planting and SRC in urban greenspaces in a typical UK city. Assessment of carbon accumulation in urban tree‐planting programmes is constrained by the limited availability of urban‐specific tree growth data. Our models mostly used growth rates reported for Europe (67%) (Table S4) and North America (13%) (Table S4). Urban‐specific growth rates only accounted for 4% of those used, reflecting the limited availability of these data. Most growth rates were derived from community woodland (24%), forestry (22%) and ex‐agricultural (16%) sites. The application of natural forest system allometric relationships to urban forests is commonplace (Timilsina *et al*. 2014), but potentially inaccurate. However, our use of averaged equations is one method of constraining errors in biomass estimates (McHale *et al*. 2009).

Fossil fuel carbon emissions occur in the nursery‐raising, transport, and planting of new trees and their subsequent maintenance (Nowak & Crane 2002; Strohbach *et al*. 2012; McPherson & Kendall 2014). These emissions are very context dependant. In the Million Trees Los Angeles Programme which covers an area of 1022 km^2^, McPherson & Kendall (2014) estimate that 6·8 kg of fossil fuel carbon is required to grow and plant each tree, mainly through use of oil in transport. In the more compact UK cities, these carbon costs are likely to be much lower. The modelled fitting of trees to suitable‐sized patches in our study results in low planting densities that will minimize the need for maintenance over 25 years. Furthermore, a comparable study of urban tree planting found the majority of trees did not need pruning (Russo *et al*. 2014), and McPherson & Kendall (2014) suggest urban tree maintenance is only about 3% of the net reduction in CO_2_ due to tree planting arising from sequestration into biomass and avoided fossil fuel carbon emissions where harvest biomass is used as a biofuel.

If our findings in Leicester are representative of the 6·8% of the UK that is urban area (Davies *et al*. 2011a), 15% of this land is suitable for combined planting of SRC and trees, suggesting that these areas hold the potential for reducing fossil fuel carbon emissions and increasing tree carbon sequestration by a total of over 7 480 000 tonnes carbon over 25 years nationally. This is a first approximation, assuming SRC is used to substitute natural gas in domestic heating, and is based on 10·3 odt ha^−1^ year^−1^ SRC yield (Aylott *et al*. 2010), rather than the 6 odt ha^−1^ year^−1^ value of Strohbach *et al*. (2012). In Leicester, soil quality data (Edmondson *et al*. 2011, 2012, 2014) justify the higher yield value. More definitive estimates of carbon savings require the tree and SRC yields on typical urban soils and landscapes to be determined, and the areas of urban land suitable for planting to be determined nationally.

Short‐rotation coppice biofuel production requires fossil fuel energy use by machinery for planting, management, harvesting and processing, resulting in carbon emissions estimated to be *c. *22% of the total global warming potential of SRC biofuel in the Mediterranean (Esteban *et al*. 2014). These components have been estimated for UK SRC production by Defra (2009) and are taken into account in our calculations of avoided carbon emissions, but are not based on urban grown SRC. In an urban context, data are required on land‐use change effects on other greenhouse gasses such as N_2_O (Don *et al*. 2012) and a life cycle assessment made of the transport and processing activities (St Clair, Hillier & Smith 2008; Holtsmark 2013). Local production and consumption will minimize transport emissions, estimated to be 11·5% of the global warming potential of SRC biofuel production in a Spanish case study (Esteban *et al*. 2014), increasing the economic viability for district energy schemes (Climate East Midlands 2012).

To meet the UK government target of 15% of all energy and 30% of electricity demand to come from renewable sources by 2020 (DECC 2009), Aylott *et al*. (2010) calculate 0·8 million ha would be required if met by SRC production. To achieve the 7·5 million odt required, all grade 5 and 97% of grade 4 agricultural land across England would be needed to avoid the best quality land. SRC production across England from 2010 to 2011 ranged from 2600 to 2700 ha (Defra 2013), indicating low acceptance of SRC by farmers. Our modelling suggests it is possible to add over 20% to the current UK SRC output by utilizing urban sites within Leicester alone. Assuming Leicester is not unique, our findings underline the untapped potential for SRC across UK urban areas.

The greatest potential for an enhanced urban carbon sequestration strategy is on the urban fringe, comprising predominantly public and mixed ownership land that can be used for tree planting or SRC. However, changed greenspace management over large areas of the city has implications for existing and future provision of ecosystem services. Urban tree planting is recognized to improve local provision of ecosystem services in ways that can positively influence local climate, carbon cycles and energy use (Davies *et al*. 2011b; Nowak *et al*. 2013a). The establishment of SRC would allow for increases in pollutant interception, microclimate amelioration, soil stabilization, visual amenity additions to heterogeneous urban areas and provide graded edges to forested areas (Wiström *et al*. 2015). However, SRC could negatively impact local ecosystem services potentially restricting public access to greenspaces and may have low public acceptance in some areas owing to the episodic aesthetic contrasts between dense mature coppice and recently harvested stools (Nielsen & Møller 2008). It is important that factors such as these are taken into consideration when selecting suitable sites for any energy crop (Aylott *et al*. 2010; Bullock *et al*. 2011). Plantations on transport route embankments may have noise reduction and pollution interception benefits, although the need for buffer zones and access for harvesting and management may ultimately exclude such sites. This highlights the importance of identifying competing interests of stakeholders, as conflicts may arise if single ecosystem services are promoted in isolation to the wider consequences (Bullock *et al*. 2011). Large areas of many cities are former industrial and derelict building, brownfield sites that are often contaminated, requiring expensive remediation before redevelopment. Such sites naturally support invading pioneer trees and could support SRC, with the added benefit of soil phytoremediation (French *et al*. 2006) although, when burning biomass, appropriate filters would need to be used (Zhao *et al*. 2014).

In conclusion, this study highlights the potential of urban greenspace for enhanced carbon management through SRC and tree planting. Carbon sequestration benefits from tree planting would continue well beyond the 25‐year scope of this study, as older trees disproportionately contribute to carbon storage (Davies *et al*. 2011b). In contrast, the benefits from fossil fuel replacement by SRC are realized much sooner, with just one mid‐sized city having the potential to add over 20% to UK production of this biomass fuel in about a decade. Even if cities across the UK only implemented a portion of the combined management approach suggested in this study, the potential for increased SRC production could reduce demand for high‐quality agricultural land to be used for biofuel production and its associated loss of food production (Renewable Fuels Agency 2008), with potential economic and societal benefits. Local authorities are central to national efforts to cut greenhouse gas emissions and need to encourage the use of urban spaces to assist in meeting the 80% reduction in CO_2_ emissions by 2050 target (UK Parliament 2008) and the EU target of 20% renewable energy by 2020 (DTI, DFT & DEFRA, 2007). The development of biomass energy sources close to large populations and encouragement of landowners (public and private) to increase carbon sequestration across a city should be part of climate change mitigation policies of city councils.

## Data accessibility

Garden Tree Survey sites and data: doi: 10.5061/dryad.j25t0 (McHugh *et al*. 2015).

Existing and modelled above‐ground carbon values: doi: 10.5061/dryad.j25t0.

Modelled carbon assimilation values: doi: 10.5061/dryad.j25t0.

## Supporting information


**Fig. S1.** Private land ownership tree planting model.
**Fig. S2.** Public land and mixed ownership land tree planting model.
**Table S1.** Mature canopy and root spread diameters of the study area tree population, based on previous published values.
**Table S2.** Land cover types and designated areas not suitable for urban carbon management by tree or SRC planting.
**Table S3.** Annual growth rates of trees used in the modelling approaches.
**Table S4.** Species, genus and family specific allometric equations used to calculate above‐ground biomass (kg) of trees.
**Appendix S1.** Calculations of biomass energy substitution by SRC biomass.
**Appendix S2.** Carbon‐offsetting potential of domestic boilers converting from fossil fuel methane to use of modelled potential production of SRC wood‐chip biomass in Leicester.
