# Supplementary material for: Modelling short‐rotation coppice and tree planting for urban carbon management – a citywide analysis
Source: J Appl Ecol. 2015 Jul 16;52(5):1237–45. doi: 10.1111/1365-2664.12491 (PMC4975693; doi:10.1111/1365-2664.12491)
Supplement: Supplementary file 1 — Fig. S1. Private land ownership tree planting model. Fig. S2. Public land and mixed ownership land tree planting model. Table S1. Mature canopy and root spread diameters of the study area tree population, based on previous published values. Table S2. Land cover types and designated areas not suitable for urban carbon management by tree or SRC planting. Table S3. Annual growth rates of trees used in the modelling approaches. Table S4. Species, genus and family specific allometric equations used to calculate above‐ground biomass (kg) of trees. Appendix S1. Calculations of biomass energy substitution by SRC biomass. Appendix S2. Carbon‐offsetting potential of domestic boilers converting from fossil fuel methane to use of modelled potential production of SRC wood‐chip biomass in Leicester. [file JPE-52-1237-s001.docx]

**Supplementary Information**

***Modelling short rotation coppice (SRC) and tree planting for urban carbon management***

***– a city-wide analysis***

by Nicola McHugh, Jill L. Edmondson, Kevin J. Gaston, Jonathan R. Leake & Odhran S. O’Sullivan

**Table S1** Mature canopy and root spread diameters of the study area tree population, based on previous published values.

| Species / Genus | Tree size | Max height (m)^a^ | Max Urban height (m)^b^ | Crown spread (m)^c^ | Root spread (%)^d^ | Root spread (m)^c^ |
| --- | --- | --- | --- | --- | --- | --- |
| *Acer plantanoides* | large | 30 |  | 18 | 90 | 16 |
| *Acer psuedoplatanus* | large | 37 | 28 | 18 | 90 | 16 |
| *Aesculus hippocastanum* | large | 37 | 28 | 20 | 90 | 18 |
| *Carpinus betulus* | large | 30 | 18 | 16 | 75 | 12 |
| *Chamaecyparis lawsoniana* | large | 42 |  | 8 | 90 | 7 |
| *Cupressocyparis leylandii* | large | 30 |  | 14 | 90 | 13 |
| *Fagus sylvatica* | large | 46 | 30 | 20 | 90 | 18 |
| *Pinus ponderosa* | large | 40 | 20 | 12 | 75 | 9 |
| *Pinus sylvestris* | large | 40 | 20 | 12 | 90 | 11 |
| *Platanus x hispanica* | large | 48 | 30 | 18 | 90 | 16 |
| *Populus nigra 'Italica'* | large | 38 |  | 8 | 90 | 7 |
| *Populus tremula* | large | 30 |  | 8 | 90 | 7 |
| *Populus x canadenis* | large | 38 |  | 20 | 90 | 18 |
| *Prunus avium* | large | 31 | 18 | 16 | 50 | 8 |
| *Quercus robur* | large | 42 | 22 | 20 | 90 | 18 |
| *Sorbus aucuparia* | large | 20 | 9 | 12 | 50 | 6 |
| *Thuja plicata* | large | 45 |  | 16 | 90 | 14 |
| *Tilia x europaea (vulgaris)* | large | 44 | 30 | 16 | 90 | 14 |
| *Abies spp.* | large | 35 | 12 | 12 |  |  |
| *Alnus spp.* | large | 25 | 15 | 14 | 75 | 11 |
| *Betula spp.* | large | 28 | 17 | 14 | 50 | 7 |
| *Cedrus spp.* | large |  |  | 16 | 90 | 14 |
| *Chamaecyparis spp.* | large | 40 | 24 | 12 | 90 | 11 |
| *Cupressus spp.* | large | 40 | 24 | 12 | 90 | 11 |
| *Eucalyptus spp.* | large | 35 |  | 16 | 75 | 12 |
| *Fraxinus spp.* | large | 41 | 17 | 18 | 90 | 16 |
| *Ilex spp.* | large | 23 |  | 8 | 75 | 6 |
| *Salix spp.* | large | 32 | 18 | 14 | 90 | 13 |
| *Ulmus spp.* | large | 40 |  | 18 | 75 | 14 |
| *Acer campestre* | small | 30 | 18 | 12 | 50 | 6 |
| *Betula pendula 'Youngii'* | small | 28 | 17 | 8 | 50 | 4 |
| *Corylus avellana* | small | 15 | 8 | 6 | 75 | 5 |
| *Crataegus monogyna* | small | 16 | 9 | 8 | 75 | 6 |
| *Eriobotrya japonica* | small | 5 | 4 | 4 | 50 | 2 |
| *Laburnum vulgare* | small | 10 |  | 6 | 75 | 5 |
| *Malus sylvestris* | small | 12 | 7 | 5 | 50 | 3 |
| *Prunus cerasifera* | small | 15 |  |  | 50 |  |
| *Prunus domestica* | small | 12 | 8 | 6 | 50 | 3 |
| *Prunus serrulata* | small | 8 | 4 | 4 | 50 | 2 |
| *Prunus spinosa* | small | 10 | 4 | 3 | 50 | 1 |
| *Prunus x schmitii* | small | 8 | 4 | 4 | 50 | 2 |
| *Pyrus communis* | small | 20 |  |  | 50 |  |
| *Sambucus nigra* | small | 10 | 4 | 3 | 75 | 2 |
| *Buddleia spp.* | small | 4 | 4 | 3 | 75 | 2 |
| *Ceonothus spp.* | small | 8 | 4 | 3 | 75 | 2 |
| *Cephalotaxus spp.* | small | 10 | 4 | 4 | 75 | 3 |
| *Cornus spp.* | small | 10 |  | 6 | 75 | 5 |
| *Ficus spp.* | small | 13 | 4 | 3 | 75 | 2 |
| *Laurus spp.* | small | 20 |  | 5 | 75 | 4 |
| *Magnolia spp.* | small | 15 |  | 6 | 75 | 5 |
| *Malus spp.* | small |  | 8 | 8 | 50 | 4 |
| *Sorbus spp.* | small | 12 | 4 | 4 | 50 | 2 |
| *Syringa spp.* | small | 4 | 3 | 3 | 75 | 2 |

Notes: Only species with greater than one individual within a sample of 1300 trees across Leicester were included in models (a total of 68 species). Data on canopy spread and root extent at maturity could only be found for 53 species, in the other 15 cases genus-level information has been presented.

^a^ Hodge & White 1990; RHS 2014.

^b^ Hodge & White 1990.

^c^ Hodge & White 1990; Gruffyd 1987.

^d^ Root spread values are expressed as a percentage of mean crown diameter based on Gruffyd 1987.

**Table S2** Landcover types and designated areas not suitable for urban carbon management by tree or SRC planting

| Class | Excluded areas | Data source |
| --- | --- | --- |
| Water and infrastructure | rivers, lakes, ponds, roads, tracks, hard standing, above and below utility lines | OS MasterMap; OS VectorMap; Infoterra LandBase |
| Designated areas | Sites of Special Scientific Interest, National and Local Nature Reserves, Special Areas of Conservation, Special Protection Areas, Ramsar Sites, Registered Common Land, Public Rights of Way (PROW) or within 5 m of PROW. | Natural England; Leicester City Council |
| Cultural and amenity areas | Scheduled Monuments, Registered Battlefields and World Heritage Sites, Sports facilities (public ownership land). | English Heritage; Leicester City Council |
| Natural habitats and landcover | Ancient Woodland Inventory, National Forestry Inventory, lowland heath, lowland calcareous grassland, lowland dry acid grassland, lowland meadow sites, within 10 m of woodland, areas of existing shrub, tall shrub and trees (private ownership land), agricultural land (grades 1-3). | Natural England; Forestry Commission; LandBase |
| Land subject to existing grants | Countryside Stewardship Agreement areas, Environmental Stewardship areas, Organic Farming Scheme areas, Woodland Grant Scheme areas. | Natural England; Forestry Commission |
|  |  |  |
| Specific to SRC | Site access areas for vehicles (3m buffer around each site), within 10 m of neighbouring land or residential property, individual plots less than 0.5 ha. | OS MasterMap |

**Table S3** Annual growth rates of trees used in the modelling approaches.

| Species / Genus | Equation type | Annual Growth cm/yr^-1^ | Origin of growth rate | | Model type | References |
| --- | --- | --- | --- | --- | --- | --- |
|  |  |  | Landcover type | Region |  |  |
| *Acer campestre* | Species | 0.37 | community woodland | UK | public land | Willoughby *et al.* 2007 |
| *Acer plantanoides* | Species | 0.74 | ex-agricultural | UK | public land | Willoughby *et al.* 2007, Wood 2010 |
| *Acer psuedoplatanus* | Species | 0.15 | forest | North America | private land | Teck & Hilt 1991 |
| *Alnus glutinosa* | Species | 0.60 | community woodland | UK | public land | Willoughby et al. 2007 |
| *Betula pendula* | Species | 0.70 | community woodland | UK | private and public land | Willoughby *et al.* 2007 |
| *Cornus sanguinea* | Species | 0.24 | community woodland | UK | public land | Teck & Hilt 1991, Willoughby *et al.* 2007 |
| *Corylus avellana* | Species | 0.21 | community woodland | UK | private and public land | Willoughby *et al.* 2007 |
| *Crataegus monogyna* | Species | 0.32 | community woodland | UK | private and public land | Willoughby *et al.* 2007 |
| *Cupressocyparis leylandii* | Species | 1.17 | community woodland | UK | private and public land | Willoughby *et al.* 2007 |
| *Fagus sylvatica* | Species | 0.51 | ex-agricultural | UK | private and public land | Willoughby *et al.* 2007 |
| *Fraxinus excelsior* | Species | 0.55 | community woodland | UK | private and public land | Willoughby *et al.* 2007 |
| *Laburnum vulgare* | Species | 0.69 | ex-agricultural | UK | private land | Willoughby *et al.* 2007 |
| *Pinus ponderosa* | Species | 1.09 | urban | North America | public land | Wood 2010 |
| *Platanus x hispanica* | Species | 0.65 | ex-agricultural | UK | public land | Willoughby *et al.* 2007 |
| *Populus tremula* | Species | 0.85 | community woodland | UK | public land | Willoughby *et al.* 2007 |
| *Populus x canadenis* | Species | 1.36 | ex-agricultural | UK | public land | Willoughby *et al.* 2007 |
| *Prunus avium* | Species | 0.67 | multiple | UK | private and public land | Willoughby *et al.* 2007 |
| *Prunus cerasifera* | Species | 0.42 | forest | North America | private land | Teck & Hilt 1991 |
| *Pyrus communis* | Species | 1.10 | ex-agricultural | UK | private land | Willoughby *et al.* 2007 |
| *Quercus robur* | Species | 0.58 | ex-agricultural | UK | public land | Willoughby *et al.* 2007 |
| *Sambucus nigra* | Species | 0.40 | multiple | UK | private and public land | Atkinson & Atkinson 2002 |
| *Thuja plicata* | Species | 1.45 | ex-agricultural | UK | private land | Willoughby *et al.* 2007 |
| *Abies spp.* | Genus | 0.94 | urban | North America | private land | Wood 2010 |
| *Acer spp.* | Genus | 0.58 | multiple | multiple | private and public land | Teck & Hilt 1991, Willoughby *et al.* 2007, Wood 2010 |
| *Aesculus spp.* | Genus | 0.15 | forest | North America | public land | Teck & Hilt 1991 |
| *Alnus spp.* | Genus | 0.68 | multiple | UK | public land | Willoughby *et al.* 2007 |
| *Betula pubescens* | Genus | 0.90 | multiple | UK | public land | Willoughby *et al.* 2007 |
| *Carpinus betulus* | Genus | 0.16 | forest | North America | public land | Teck & Hilt 1991 |
| *Cornus spp.* | Genus | 0.24 | community woodland | UK | private land | Willoughby *et al.* 2007 |
| *Eucalyptus spp.* | Genus | 2.40 | multiple | multiple | private land | Kumar *et al.* 2010, Misra *et al.* 1998 |
| *Fraxinus ornus* | Genus | 0.44 | multiple | UK | public land | Willoughby *et al.* 2007 |
| *Ilex spp.* | Genus | 0.15 | forest | North America | private and public land | Teck & Hilt 1991 |
| *Laurus spp.* | Genus | 0.15 | forest | North America | private land | Teck & Hilt 1991 |
| *Magnolia spp.* | Genus | 0.15 | forest | North America | private land | Teck & Hilt 1991 |
| *Malus spp.* | Genus | 0.47 | multiple | multiple | private and public land | Teck & Hilt 1991, Willoughby *et al.* 2007, Wood 2010 |
| *Pinus spp.* | Genus | 1.02 | multiple | multiple | public land | Teck & Hilt 1991, Willoughby *et al.* 2007, Wood 2010 |
| *Populus spp.* | Genus | 1.10 | multiple | UK | public land | Willoughby *et al.* 2007 |
| *Prunus spp.* | Genus | 0.67 | multiple | multiple | private and public land | , Teck & Hilt 1991, Willoughby *et al.* 2007 |
| *Salix spp.* | Genus | 0.58 | community woodland | UK | private and public land | Willoughby *et al.* 2007 |
| *Sorbus spp.* | Genus | 0.65 | forest | North America | private and public land | Teck & Hilt 1991 |
| *Tilia spp.* | Genus | 0.55 | multiple | multiple | public land | Willoughby *et al.* 2007, Wood 2010 |
| *Ulmus spp.* | Genus | 0.15 | forest | North America | public land | Teck & Hilt 1991 |
| *Pinacea* | Family | 0.71 | multiple | multiple | private land | Teck & Hilt 1991, Willoughby *et al.* 2007, Wood 2010 |
| *Cupressaceae* | Family | 1.31 | multiple | UK | private and public land | Willoughby *et al.* 2007 |
| *Moraceae* | Family | 0.16 | forest | Asia | private land | Primack *et al.* 1985 |
| *Rhamnaceae* | Family | 0.32 | community woodland | UK | private land | Willoughby *et al.* 2007 |
| *Rosaceae* | Family | 0.65 | multiple | multiple | private land | Teck & Hilt 1991, Willoughby *et al.* 2007, Wood 2010 |
| *Syringa spp.* | Family | 0.44 | multiple | multiple | private land | Teck & Hilt 1991, Willoughby *et al.* 2007, Wood 2010 |
| *Taxaceae* | Family | 0.14 | arboretum | Europe | private land | Iszkuło &Boratyński 2005 |
| Deciduous^a^ |  | 0.50 | multiple | multiple | private land | Teck & Hilt 1991, Willoughby *et al.* 2007, Wood 2010 |

^a^ growth rate uses mean growth rate of all deciduous tree species which have species specific growth rates.

| Atkinson, M. D. & Atkinson, E. (2002) *Sambucus nigra L. Journal of Ecology,* **90**, 895-923.  Iszkuło, G. &Boratyński, A. (2005) Different age and spatial structure of two sponteneous subpopulations of *Taxus baccata* as a result of various intensity of colonization process. *Flora*, **200**, 195-206.  Kumar, A., Luna, R. K., Parveen & Kumar, V. (2010) Variability in growth characteristics for different genotypes of *Eucalyptus tereticornis* (SM.). *Journal of Forestry Research*, **21**, 487-491.  Misra, R. K., Turnbull, C. R. A., Cromer, R. N., Gibbons, A. K. & LaSala, A. V. (1998) Below- and above-ground growth of *Eucalyptus nitens* in a young plantation I. Biomass. *Forest Ecology and Management*, **106**, 283-293.  Primack, R. B., Ashton, P. S., Chai, P. & Lee, H. S. (1985) Growth rates and population structure of Moraceae trees in Sarawak, Malaysia. *Ecology*, **66**, 577-588.  Teck, R. M. & Hilt, D. E. (1991) *Individual-tree diameter growth model for the Northeastern United States*. Research paper NE-649, USDA Forest Service, Radnor, Pennsylvania.  Willoughby, I., Stokes, V., Poole, J., White, J.E.J & Hodge, S.J. (2007) The potential of 44 native and non-native tree species for woodland creation on a range of contrasting sites in lowland Britain. *Forestry*, **80**, 531-553. |
| --- |
| Wood, K. (2010) *Growth rates of common tree species in Westminster, Colorado.* Colorado State Forest Service, Fort Collins, Colorado. |

**Table S4** Species, genus and family specific allometric equations used to calculate aboveground biomass (kg) of trees.

| Species, genus or family level equation | Equation | DBH range (cm) / Height range (m) | Reference |
| --- | --- | --- | --- |
| *Acer saccharum* | 0.1008 (DBH)^2.5735^ | DBH 5-50 / no ht | Ter-Mikaelian & Korzukhin (1997) |
|  | 0.1532(DBH)^2.3924^ * 1.011 | DBH 1-34 / no ht |  |
|  | 0.1599(DBH)^2.3376^ * 1.010 | DBH 1-41 / no ht |  |
|  | 0.1676(DBH)^2.3646^ | DBH 4-34 / no ht |  |
|  | 0.1641(DBH)^2.4209^ *1.004 | DBH 1-50 / no ht |  |
|  | 0.1791(DBH)^2.3329^ | DBH 3-66 / no ht |  |
| *Alnus glutinosa* | 0.00079 (DBH)^2.28546^ | DBH 1-17.3 / ht 2.5-17.6 | Zianis *et al.* (2005) |
|  | 0.003090 (DBH)^2.022126^ | DBH 12.2-28.3 / ht 13-25.4 |  |
|  | 0.0859(DBH)^2.3537^ | no DBH / no ht |  |
| *Alnus incana* | 0.00030 (DBH)^2.42847^ | DBH 0.7-9.3 / ht 2-14.8 | Zianis *et al.* (2005) |
|  | 0.000499 (DBH)^2.337592^ | DBH 8.9-24.6 / ht 13-25.3 |  |
| *Betula pendula* | 0.00087 (DBH)^2.28639^ | DBH 1.8-13.7 / ht 3.2-19.9 | Zianis *et al.* (2005) |
|  | 0.2511(DBH)^2.29^ | no DBH / no ht |  |
| *Betula pubescens* | 0.00029 (DBH)^2.50038^ | DBH 0.8-8.5 / ht 2.3-12 | Zianis *et al.* (2005) |
| *Fagus sylvatica* | 0.453 (DBH)^2.139^ | DBH 5.7-62.1 / ht 9.2-33.9 | Zianis *et al.* (2005); Muukkonen & Mäkipää (2006) |
|  | 0.0798 (DBH)^2.601^ | no DBH / no ht |  |
|  | 0.1315 (DBH)^2.4321^ | DBH 4-34.5 / 6.1-18.4 |  |
|  | -1.0798 + 0.018017 * (DBH)^2^ * (height) + 0.25888 * (DBH)^2^ | DBH 9.5-56.5 / ht 9.3-22.3 |  |
|  | -2.872+2.095 * ln (DBH) + 0.678 * ln (height) | no DBH / no ht |  |
| *Fraxinus excelsior* | (ln 4.0043 + 3.0495 * ln (DBH))/1000 | DBH 0-8.1 / ht 0.26-9.1 | Blujdea et al, (2012) |
| *Pinus nigra spp. Laricio* | -3.5712 + 0.014429 * (DBH)^2^ * (height) + 0.068047 *(DBH)^2^ | DBH 8.9-35.9 / ht 5.6-20.9 | Muukkonen & Mäkipää (2006) |
| *Pinus sylvestris* | (200.87186*(DBH)^2^+124.6808 ((DBH)^2^-49))/1000 | DBH 7-20 / no ht | Muukkonen & Mäkipää (2006); Zianis *et al.* (2005) |
|  | 0.1182 (DBH)^2.3281^ | DBH 2-16 / ht 4-11 |  |
|  | (0.981+ 2.289 * log (π (DBH)))/1000 | no DBH / no ht |  |
|  | -0.73626 + 0.018465 * (DBH)^2^ * (height) | DBH 8.4-40.6 / ht 6.4-20.8 |  |
|  | 0.0146 ((DBH)+1)^2.3868+-0.0618*log (DBH)^ * (height) 0.8581 | DBH 2-16 / ht 4-11 |  |
| *Populus tremula* | 0.0519 (DBH)^2.545^ | DBH 13.2-33 / ht 15.9-24.7 | Zianis et al. (2005) |
|  | (1.46*10^-4^)*(DBH)^2.6035333^ | DBH 1.9-9.2 / ht 3.6-15.8 |  |
| *Quercus ilex* | 0.2179 (DBH)^2.0513^ | DBH 5-20 / no ht | Zianis et al. (2005) |
|  | -0.6165+0.03582 (DBH) ^2^ * (height) | DBH 4.5-26.1 / ht 6-16 |  |
|  | -0.854+2.413 log (DBH) | DBH 5.3-24.4 / ht 4.4-12.8 |  |
|  | -0.902+2.433 log (DBH) | DBH 5.3-24.4 / ht 4.4-12.8 |  |
|  | -0.568+1.953 log (DBH) + 0.029 (height) | DBH 5.3-24.4 / ht 4.4-12.8 |  |
|  | 0.2313*(DBH)^2.2662^ | no DBH / no ht |  |
| *Sorbus aucuparia* | 0.0634 * (DBH)^2.1552^ * (height)^0.2877^ | DBH 2.1-29.8 / ht 2.1-11.6 | Snorrason & Einarsson (2006) |
| *Abies spp.* | 0.0877*(DBH)^2.4017^ | DBH 3-51 / no ht | Ter-Mikaelian & Korzukhin (1997) |
|  | 0.0690*(DBH)^2.4975^ * 1.008 | DBH 3-40 / no ht |  |
|  | 0.2575*(DBH)^2.0543^ * 1.039 | DBH 3-40 / no ht |  |
|  | 0.1746*(DBH) ^2.1555^ | DBH 0-36 / no ht |  |
|  | 0.1598*(DBH)^2.1283^ * 1.030 | DBH 2-32 / no ht |  |
|  | 0.0705*(DBH)^2.4970^ * 1.015 | DBH 4-34 / no ht |  |
| *Acer spp.* | 0.1791*(DBH)^2.3329^ | DBH 3-66 / no ht | Ter-Mikaelian & Korzukhin (1997) |
|  | 0.1262*(DBH)^2.3804^ | DBH 3-66 / no ht |  |
|  | 0.1641*(DBH)^2.4209^ *1.004 | DBH 1-50 / no ht |  |
|  | 0.1008*(DBH)^2.5735^ | DBH 5-50 / no ht |  |
|  | 0.0910*(DBH)^2.5080^ | DBH 5-50 / no ht |  |
|  | 0.1789*(DBH)^2.3340^ *1.007 | DBH 10-52 / no ht |  |
| *Aesculus spp.* | 2.6572 + 0.9451 * ln (CBH) | no DBH/no ht | Adhikari et al 1995 |
| *Alnus spp.* | 0.00079 *(DBH)^2.28546^ | DBH 1-17.3 / ht 2.5-17.6 | Zianis et al. (2005) |
|  | 0.00030 * (DBH)^2.42847^ | DBH 0.7-9.3 / ht 2-14.8 |  |
|  | 0.000499 * (DBH)^2.337592^ | DBH 8.9-24.6 / ht 13-25.3 |  |
| *Cupressus spp.* | 0.2498*(DBH)^2.1118^ *1.008 | DBH 18-60 / no ht | Ter-Mikaelian & Korzukhin (1997) |
| *Eucalyptus spp.* | -1.762 + 2.2644 * ln (DBH) | DBH 4-25 / no ht | Zianis et al. (2005) |
| *Fraxinus spp.* | (ln 4.0043 + 3.0495 * ln (DBH))/1000 | DBH 0-8.1 / ht 0.26-9.1 | Ter-Mikaelian & Korzukhin (1997); Blujdea *et al*. (2012) |
|  | 0.1634*(DBH)^2.3480^ | DBH 4-32 / no ht |  |
|  | 0.1063*(DBH)^2.4798^ | DBH 5-50 / no ht |  |
|  | 0.1535*(DBH)^2.3213^ * 1.010 | DBH 1-28 / no ht |  |
|  | 0.1634*(DBH)^2.3480^ | DBH 4-32 / no ht |  |
| *Ilex spp.* | 2.0062 * (DBH) -0.7229 | no DBH / no ht | Adhikari *et al.* (1995); Aboal *et al.* (2005) |
|  | 0.7752 + 0.906 * ln (CBH) | no DBH/no ht |  |
| *Laurus spp.* | 2.4392 * (DBH) -2.1484 | no DBH / no ht | Aboal *et al.* (2005) |
| *Pinus spp.* | -0.73626 + 0.018465 *(DBH)^2^ * (height) | DBH 8.4-40.6 / ht 6.4-20.8 | Zianis *et al.* (2005); Muukkonen & Mäkipää (2006) |
|  | -3.5712 + 0.014429 * (DBH)^2^ * (height) + 0.068047 * (DBH)^2^ | DBH 8.9-35.9 / ht 5.6-20.9 |  |
|  | 0.0146 * ((DBH)+1)^2.3868+-0.0618 * log(DBH)^ *(height)^0.8581^ | DBH 2-16 / ht 4-11 |  |
|  | (200.87186 * (DBH)^2^ + 124.6808 *((DBH)^2^-49))/1000 | DBH 7-20 / no ht |  |
|  | 0.1182 * (DBH)^2.3281^ | DBH 2-16 / ht 4-11 |  |
|  | (0.981 + 2.289 * log(π*(DBH)))/1000 | no DBH / no ht |  |
| *Populus spp.* | 0.7646 *(DBH) / 2.54 + 2.5655 * ln (DBH) / 2.54) * 0.4535923 | DBH 6.45-129.03 / no ht | Ter-Mikaelian & Korzukhin (1997); Jenkins *et al.* (2004); Zianis *et al.* (2005) |
|  | 0.0785*(DBH)^2.4981^ | DBH 3-45 / no ht |  |
|  | 0.0637*(DBH)^2.6087^ | DBH 3-51 / no ht |  |
|  | 0.0519 * (DBH)^2.545^ | DBH 13.2-33 / ht 15.9-24.7 |  |
|  | 1.46*10^-4^ * (DBH)^2.6035333^ | DBH 1.9-9.2 / ht 3.6-15.8 |  |
|  | 0.0717 * (DBH)^1.8322^ * (height)^0.6397^ | DBH 4.6-34 / ht 4.6-20.7 |  |
| *Quercus spp.* | 0.0945*(DBH) ^2.5030^ | DBH 5-40 / no ht | Ter-Mikaelian & Korzukhin (1997); Zianis *et al.* (2005) |
|  | 0.0554*(DBH)^2.7276^ | DBH 5-50 / no ht |  |
|  | 0.1130*(DBH)^2.4572^ | DBH 5-50 / no ht |  |
|  | 0.1241*(DBH)^2.4395^ | DBH 5-40 / no ht |  |
|  | 0.0579*(DBH)^2.6887^ | DBH 5-50 / no ht |  |
|  | -0.883 + 2.140 * ln(DBH) | no DBH / no ht |  |
| *Salix spp.* | 0.0348 * (DBH)^1.9123^ * (height)^0.8904^ | DBH 2.4-23.9 / ht 1.9-8.8 | Ter-Mikaelian & Korzukhin (1997); Snorrason & Einarsson (2006) |
|  | 0.0616*(DBH)^2.5094^ | DBH 4-20 / no ht |  |
|  | 0.1619*(DBH)^2.0552^ | DBH 3-24 / no ht |  |
| *Sorbus spp.* | 0.0634 * (DBH)^2.1552^ * (height)^0.2877^ | DBH 2.1-29.8 / ht 2.1-11.6 | Snorrason & Einarsson (2006) |
| *Thuja spp.* | 0.1148*(DBH)^2.1439^ *1.010 | DBH 2-30 / no ht | Ter-Mikaelian & Korzukhin (1997) |
|  | 0.0910*(DBH)^2.234^ *1.008 | DBH 4-31 / no ht |  |
|  | 0.2305*(DBH)^1.9269^ | DBH 3-51 / no ht |  |
| *Tilia spp.* | 0.0617*(DBH)^2.5328^ | DBH 5-50 / no ht | Ter-Mikaelian & Korzukhin (1997); Zianis et al. (2005) |
|  | 0.0872*(DBH)^2.3539^ | DBH 4-47 / no ht |  |
|  | -2.6788 + 2.4542 * ln(DBH) | DBH 3.2-15 / no ht |  |
| *Ulmus spp.* | (0.0825*(DBH)^2.468^ *1.011 | DBH 4-29 / no ht | Ter-Mikaelian & Korzukhin (1997) |
| *Betulaceae* | 0.00079 * (DBH)^2.28546^ | DBH 1-17.3 / ht 2.5-17.6 | Zianis et al. (2005) |
|  | 0.00030 * (DBH)^2.42847^ | DBH 0.7-9.3 / ht 2-14.8 |  |
|  | 0.000499 * (DBH)^2.337592^ | DBH 8.9-24.6 / ht 13-25.3 |  |
|  | 0.00087 * (DBH)^2.28639^ | DBH 1.8-13.7 / ht 3.2-19.9 |  |
|  | 0.00029 * (DBH)^2.50038^ | DBH 0.8-8.5 / ht 2.3-12 |  |
| *Cupressaceae* | 0.2498*(DBH)^2.1118^ *1.008 | DBH 18-60 / no ht | Ter-Mikaelian & Korzukhin (1997) |
|  | 0.1148*(DBH)^2.1439^ *1.010 | DBH 2-30 / no ht |  |
|  | 0.0910*(DBH)^2.2340^ *1.008 | DBH 4-31 / no ht |  |
|  | 0.2305*(DBH)^1.9269^ | DBH 3-51 / no ht |  |
| *Fagaceae* | 0.113*(DBH)^2.4572^ | DBH 5-50 / no ht | Ter-Mikaelian & Korzukhin (1997); Zianis et al. (2005) |
|  | 0.1241*(DBH)^2.4395^ | DBH 5-40 / no ht |  |
|  | 0.0579*(DBH)^2.6887^ | DBH 5-50 / no ht |  |
|  | -2.872 + 2.095 * ln(DBH) + 0.678 * ln(height) | no DBH / no ht |  |
|  | 0.453 * (DBH)^2.139^ | DBH 5.7-62.1 / ht 9.2-33.9 |  |
|  | 0.1315 * (DBH)^2.4321^ | DBH 4-34.5 / 6.1-18.4 |  |
| *Moraceae* | 0.1525 * (DBH)^2.34^ | no DBH / no ht | Kenzo et al. (2009) |
|  | 0.1083 * (DBH)^2^ * (height)^0.80^ | no DBH/no ht |  |
| *Oleaceae* | 0.1634*(DBH)^2.3480^ | DBH 4-32 / no ht | Ter-Mikaelian & Korzukhin (1997) |
|  | 0.1063*(DBH)^2.4798^ | DBH 5-50 / no ht |  |
|  | 0.1535*(DBH)^2.3213^ * 1.010 | DBH 1-28 / no ht |  |
|  | 0.1634*(DBH)^2.3480^ | DBH 4-32 / no ht |  |
| *Pinaceae* | -3.5712 + 0.014429 * (DBH)^2^ * (height) + 0.068047 * (DBH)^2^ | DBH 8.9-35.9 / ht 5.6-20.9 | Ter-Mikaelian & Korzukhin (1997); Zianis *et al.* (2005); Muukkonen & Mäkipää (2006) |
|  | 0.1598*(DBH)^2.1283^ *1.030 | DBH 2-32 / no ht |  |
|  | 0.0705*(DBH)^2.4970^ *1.015 | DBH 4-34 / no ht |  |
|  | -43.13 + 2.25 * (DBH) + 0.452 * (DBH)^2^ | DBH 17-39 / no DBH |  |
|  | 0.1182 * (DBH)^2.3281^ | DBH 2-16 / 4-11 |  |
|  | (0.981 + 2.289 * log(π * (DBH)))/1000 | no DBH / no ht |  |
| *Rosacaea* | 1.1981 + 1.5876 * log (DBH)^2^ / 1000 | DBH 0.37-6.83 / no ht | Ter-Mikaelian & Korzukhin (1997); Jenkins *et al.* (2003); Snorrason & Einarsson (2006) |
|  | 0.0634 * (DBH)^2.1552^ * (height)^0.2877^ | DBH 2.1-29.8 / ht 2.1-11.6 |  |
|  | 0.0716*(DBH)^2.6174^ | DBH 5-50 / no ht |  |
|  | 0.1225*(DBH)^2.4253^ | DBH 5-40 / no ht |  |
|  | 0.2643*(DBH)^1.7102^ | DBH 3-15 / no ht |  |
|  | 0.1556*(DBH)^2.1948^ | DBH 3-24 / no ht |  |
| Coniferous | 7.295*(height)^1.395^ | no DBH / no ht | Davies *et al. (*2011) |
| Deciduous | 0.566*(height)^2.315^ | no DBH / no ht | Davies *et al.* (2011) |

Aboal, J. R., Arévalo, J. R. & Fernández, A. (2005) Allometric relationships of different tree species and stand above ground biomass in the Gomera laurel forest (Canary Islands). *Flora*, **200**, 264-274.

Adhikari, B. S., Rawat Y. S. & Singh, S. P. (1995) Structure and function of high altitude forests of Central Himalaya I. Dry matter dynamics. *Annals of Botany,* **75,** 237-248.

Blujdea, V. N. B., Pilli, R., Dutca, L., Ciuvat, L. & Abrudan, I. V. (2012) Allometric biomass equations for young broadleaved trees in plantations in Romania. *Forest Ecology and Management*, **264**, 172-184.

Davies, Z.G., Edmondson, J.L., Heinemeyer, A., Leake, J.R. & Gaston, K.J. (2011) Mapping an urban ecosystem service: quantifying above-ground carbon storage at a city-wide scale. *Journal of Applied Ecology,* **48**, 1125-1134.

Jenkins, J. C., Chojnacky, D. C., Heath, L. S. & Birdsey, R. A. (2003) *Comprehensive database of diameter-based biomass regressions for North American tree species,* General Technical Report NE-319, USDA Forest Service, Burlington, Vermont.

Kenzo, T., Furutani, R., Hattori, D., Kendawang, J. J., Tanaka, S., Sakurai, K. & Ninomiya, I. (2009) Allometric equations for accurate estimation of above-ground biomass in logged-over tropical rainforests in Sarawak, Malaysia. *Journal of Forestry Research*, **14**, 365-372.

Muukkonen, P. & Mäkipää, R. (2006) Biomass equations for European trees: Addendum. *Silva Fennica*, **40**, 763-773.

Snorrason, A. & Einarsson, S. F. (2006) Single-tree biomass and stem volume functions of eleven tree species used in Icelandic forestry. *Icelandic Agricultural Sciences*, **19**, 15-24.

Ter-Mikaelian, M. T. & Korzukhin, M. D. (1997) Biomass equations for sixty-five North American tree species. *Forest Ecology and Management*, **97**, 1-24.

Zianis, D., Muukkonen, P., Mäkipää, R. & Mencuccini, M. (2005) Biomass and stem volume equations for tree species in Europe. *Silva Fennica*, **Monographs 4**, 1-63.

**Figure S1** Private land ownership tree planting model

Sufficient distance from impervious?

Landcover

Pervious surface

Existing tree canopy

Impervious surface

Overlay of surfaces

Plantable area?

Plant tree in centre

Project mature tree crown area

Overlap?

Add tree to database

Conclude planting?

Stop planting and finalise database

Future tree canopy

Updated pervious surface availability

Eliminate

N

N

Eliminate

Y

Eliminate

N

Y

Y

N

Y

Urban and age specific mortality rate applied

Tree survives?

Y

Remove

N

Re-planting rate applied

Additional trees added to database

**Tree mortality and re-planting**

N

Y

Appropriate growth rate?

Y

N

Use same rate

Identify appropriate rate

**Tree growth**

Garden tree population

Species with >1 individual

Exclude species

Growth rate applied

Y

Biomass and carbon calculated per tree

Growth year 1 to 24?

N

Finalise private land ownership tree database

**Figure S2** Public land and mixed ownership land tree planting model

Urban and age specific mortality rate applied

Tree survives?

Y

Remove

N

Re-planting rate applied

Additional trees added to database

**Tree mortality and re-planting**

N

Y

Appropriate growth rate?

Y

N

Use same rate

Identify appropriate rate

**Tree growth**

tree population

Species with >1 individual

Exclude species

Growth rate applied

Y

Overlap herbaceous landcover polygon edges?

Herbaceous landcover

Maximum tree planting grid

Project mature tree crown area

Stop planting and finalise database

N

Eliminate

Y

Add tree to database

Erase herbaceous areas where planting is allocated

Updated herbaceous landcover

Biomass and carbon calculated per tree

Growth year 1 to 24?

N

Finalise public land and mixed land ownership tree database

**Appendix S1** *Calculations of biomass energy substitution by SRC biomass*

The Biomass Energy Centre is the government information centre for the use of biomass for energy in the UK. This provides compiled data on the current efficiency of conversion of different kinds of biomass to bioenergy, and data for calculations of carbon-offsetting by use of biomass fuels to substitute for fossil fuels.

The Biomass Energy Centre (2014) published data on the quantities of wood-chip biomass from SRC that are required to supply the heating requirements domestic houses, district heating schemes and municipal buildings. We converted the raw biomass data (30% moisture content) into dry biomass (x 0.70) and into carbon content (x 0.46, the proportion of deciduous tree wood that is carbon).

A typical domestic House (20 MWh_th_), would use 5.7 tonnes wood chips per year with 30% moisture content = 3.99 tonnes oven dried biomass.  This has a carbon content of 46% so the biomass C demand per house is 1.835 tonnes per year.

District Heating Scheme of 600 MWh_th_ would use 170 tonnes wood chips per year with 30% moisture content =119 tonnes oven dried biomass.  This has a carbon content of 54.7 tonnes per year.

Municipal Buildings of 1000 MWh_th_ would use 290 tonnes wood chips per year with 30% moisture content =203 tonnes oven dried biomass.  This has a carbon content of 93.38 tonnes per year.

The modelled potential production of SRC carbon in Leicester of 2873.9 tonnes of C per year (the total production of 71,847 tonnes averaged over 25 years) can be divided by these carbon consumption values to indicate the average numbers of the different kinds of buildings and heating systems that could be supported per year over this period.

For houses we can supply 2873.9 tonnes of C per year / 1.835 tonnes C per year = 1566 houses provided with heating.

For district heating scheme we can supply 2873.9 tonnes of C per year / 54.7 tonnes C per year = 52 district heating schemes supplied.

For municipal buildings we can supply 2873.9 tonnes of C per year / 93.38 tonnes C per year = 30 municipal buildings supplied.

*Example award winning district energy scheme in Barnsley*

The Sheffield Road Flats in Barnsley are 166 flats heated with a 320 KW and 150 KW boiler (the smaller one used in the summer when demand is lower), (Barnsley Metropolitan Borough Council, 2006).

The boilers burn 350 tonnes of wood chips per year.  The boilers can cope with chips up to 60% moisture content.  The typical moisture content is not specified, but we assume that it is 30%, and the dry biomass has 46% carbon content. The total biomass carbon burnt = 112.7 tonnes per year.

The modelled production of SRC carbon of 2873.9 tonnes of C per year in Leicester would support 25.5 such schemes = heating to (25.5 x 166 flats) = 4233 flats. 
There are 126,923 households in Leicester (Office for National Statistics 2012) so this would permit heating of 3.3% of Leicester households.

**Appendix S2** *Carbon-offsetting potential of domestic boilers converting from fossil-fuel methane to use of modelled potential production of SRC wood-chip biomass in Leicester.*

A lifecycle analysis (Defra 2009) provides assessment of greenhouse gas emissions as CO_2_ equivalents for different kinds of biomass fuels versus oil and natural gas (see Figure 3.7 for details of SRC versus natural gas). The data includes estimates of cultivation/ extraction, processing, transport and combustion CO_2_ release of fossil-fuel carbon. It shows the greenhouse gas warming potential of burning fossil fuel methane is 206 kg CO_2_ per MWh,= 56.18 kg of C per MWh, whereas for SRC biomass chips it is 23.5 kg CO_2_ per MWh, or 6.40 kg of C per MWh. Thus for each MWh that SRC biomass substitutes methane it saves 49.78 kg of C.

Each house typically consumes 15 MWh of gas (DECC 2013) so each house switching to SRC biomass fuel would save 49.78 x 15 = 746.7 kg of fossil fuel C emissions per year.

From the calculations of biomass energy substitution for fossil fuels we established that the SRC could support the heating of 1566.16 houses. 1566.16 x 746.7 kg of saved fossil fuel C = 1169.45 tonnes emissions saved per year. Over 25 years this amounts to a total of 29,236 tonnes of C saved from emissions.

We found that tree planting in the remaining areas not suitable to SRC would sequester 4,183 tonnes of carbon in biomass in Leicester over 25 years, so that the total potential reduction of carbon in the atmosphere from the combined tree and SRC planting and management is 33,419 tonnes. This assumes that existing stocks of carbon in herbaceous vegetation in the areas planted by trees would not change- a reasonable assumption given the relatively low density of tree planting.

Note that these estimates are subject to uncertainties regarding the types of fuel that SRC may substitute for over the next 25 years, and the efficiency of the fossil fuel and biomass boilers that are used over this period of time. Furthermore, although we have assumed that the SRC biomass would be used as a fuel, it could be used in a wide variety of products such as fibreboard, barbeque charcoal (where it might substitute for tropical forest tree felling), biochar additions to soil, etc. Each of these different types of use would have different context- and technology- dependent carbon-offsetting potentials.

**References**

Barnsley Metropolitan Borough Council (2006). District heating from local tree waste. Technical report. [www.ashden.org/winners/barnsley](http://www.ashden.org/winners/barnsley) (last accessed April 2015).

Biomass Energy Centre (2014) *Biomass heating of buildings of different sizes*. [www.biomassenergycentre.org.uk/portal/page?_pageid=75,163211&_dad=portal&_schema=PORTAL](http://www.biomassenergycentre.org.uk/portal/page?_pageid=75,163211&_dad=portal&_schema=PORTAL) (last accessed April 2015).

DECC (2013) <https://www.gov.uk/government/statistics/energy-consumption-in-the-uk> (last accessed April 2015). See Ch. 3 Domestic Data Tables, Table 3.07:

Defra (2009), *Carbon factor for wood fuels for the Supplier Obligation.* AEA Final Report to Department for Environment Food and Rural Affairs, London) See Figure 3.7

Office for National Statistics 2012 <http://www.ons.gov.uk/ons/rel/mro/news-release/census-result-shows-increase-in-population-of-the-east-midlands/censuseastmidnr0712.html> (last accessed April 2015).
